# Association of Postpartum Maternal Morbidities with Children's Mental, Psychomotor and Language Development in Rural Bangladesh

**DOI:** 10.3329/jhpn.v30i2.11313

**Published:** 2012-06

**Authors:** J.D. Hamadani, F. Tofail, A. Hilaly, F. Mehrin, S. Shiraji, S. Banu, S.N. Huda

**Affiliations:** ^1^icddr,b, GPO Box 128, Dhaka 1000, Bangladesh; ^2^Institute of Nutrition and Food Science, University of Dhaka, Dhaka 1000, Bangladesh

**Keywords:** Anaemia, Child development, Cognitive development, Descriptive studies, Morbidity, Psychomotor development, Bangladesh

## Abstract

Little is known from developing countries about the effects of maternal morbidities diagnosed in the postpartum period on children's development. The study aimed to document the relationships of such morbidities with care-giving practices by mothers, children's developmental milestones and their language, mental and psychomotor development. Maternal morbidities were identified through physical examination at 6-9 weeks postpartum (n=488). Maternal care-giving practices and postnatal depression were assessed also at 6-9 weeks postpartum. Children's milestones of development were measured at six months, and their mental (MDI) and psychomotor (PDI) development, language comprehension and expression, and quality of psychosocial stimulation at home were assessed at 12 months. Several approaches were used for identifying the relationships among different maternal morbidities, diagnosed by physicians, with children's development. After controlling for the potential confounders, maternal anaemia diagnosed postpartum showed a small but significantly negative effect on children's language expression while the effects on language comprehension did not reach the significance level (p=0.085). Children's development at 12 months was related to psychosocial stimulation at home, nutritional status, education of parents, socioeconomic status, and care-giving practices of mothers at six weeks of age. Only a few mothers experienced each specific morbidity, and with the exception of anaemia, the sample-size was insufficient to make a conclusion regarding each specific morbidity. Further research with a sufficient sample-size of individual morbidities is required to determine the association of postpartum maternal morbidities with children's development.

## INTRODUCTION

Growth, development, and behaviour of children and, thus, overall future national productivity depend on several biological, psychosocial and economic factors, most of which are closely interrelated (1). For example, pre- and postnatal conditions and exposures of both mother and child to biological and environmental risks, influence children's development (2). More specifically, intrauterine growth retardation (IUGR) and preterm birth, which are the major determinants of neonatal mortality and morbidity, have long-term adverse consequences for health (3-5) and development of children (2). Morbidities in children associated with preterm birth and IUGR often extend to the later life, resulting in enormous physical, psychological and economic costs (6-9). Less is known, however, of the effect of maternal morbidities suffered by women in the postpartum period that could affect child development. These morbidities could be a result of intrapartum morbidities or of management at birth.

Morbidities in women before and during birth have been relatively well-documented. A systematic review of maternal morbidities suffered in pregnancy or at childbirth in reports from different countries published during 1997-2002 (10) showed that, hypertensive disorders of pregnancy (14.9%) were most frequently documented while other morbidities included haemorrhage (6.2%), premature rupture of the membrane (2.4%), perineal laceration (2.3%), and obstructed labour (1.7%). Most common outcomes relating to infants noted at birth included stillbirth (13.9%) and preterm delivery (8.2%). In Bangladesh, commonly-reported maternal morbidities during pregnancy and delivery include oedema, anaemia, proteinuria, high blood pressure, pre-eclampsia, premature rupture of the membrane, and bleeding (11). In this series, we report that about 10% of all women giving birth suffered from severe or less-severe maternal complications at birth (12)—primarily dystocia, haemorrhage, or pre-eclampsia/eclampsia. Those with severe complications experience a higher rate of perinatal deaths. Morbidities suffered in the postpartum period are far less studied. Ferdous *et al*. (13) reported in this special issue that moderate anaemia was one of the major outcomes for women who had suffered from an acute morbidity during delivery; other postpartum problems, e.g. prolapse, hernias, and haemorrhoids, were mostly mild (13).

The relationship of any of the maternal morbidities, originating either during the intrapartum or postpartum period, with development of children in Bangladesh is still unknown. It is known from an earlier study in the same area—Matlab—that women aged ≤18 years, those with poor obstetric history, undernutrition, pre-eclampsia, and jaundice, had a much higher risk of perinatal death (14). The fate of the surviving babies of these women was not reported; however, it can be hypothesized that they had a higher risk of developmental problems.

The few published papers relating to maternal illnesses and children's cognitive function are based on facility-based data and are primarily focused on morbidities in pregnancy or during the intrapartum period. Examples include newborn infants of diabetic mothers demonstrating subtle evidence of impairments in recognition memory (15) and children of epileptic mothers showing minor and major congenital anomalies (16). Undernourished mothers are at a risk of delivering infants with low birthweight (3), who suffer from several physical, economic and intellectual consequences later in life (8,9). At the same time, iron deficiency during pregnancy diminishes iron stores in the foetus (17) and is a risk factor for infant's anaemia (18), which, in turn, leads to poorer development of infants and children (9,19). Moreover, mothers with iron-deficiency anaemia during pregnancy had poorer interactions with their children (20). In addition, maternal depression and dissatisfaction with life suffered after delivery are associated with poorer development and behaviour of children (21).

We found few published reports linking the specific maternal morbidities known to kill mothers during and after delivery (e.g. haemorrhage, eclampsia, obstructed labour), or postpartum morbidities (e.g. vesicovaginal fistula, rectovaginal fistula, perineal tears, uterine prolapse) to children's development. The hypothesis for our study was that postpartum morbidities/conditions, resulting from acute maternal morbidities or even management of normal childbirth, could constrain the mother's ability to carry out appropriate care for her child and, thus, affect the child's development. In addition, little data are available on type of delivery and child development outcomes (22-24), and the existing findings are not consistent.

During 2007-2008, the maternal morbidity study in Matlab observed women from delivery to 42 days postpartum to determine the types and prevalence of complications relating to childbirth and the postpartum period. Hospital levels of severe (6.7%) and less-severe (14.8%) maternal complications among women were mostly related to dystocia (68.8%), hypertensive disorders (12.6%), haemorrhage (8.4%), anaemia (6.4%), and genital infection (3.8%) (12). A sample of these women, along with a sample drawn from women with normal vaginal delivery or a caesarean section without maternal indication, were followed up at 6-9 weeks, with a physical examination by a physician to determine postpartum morbidities. The findings included such postpartum morbidities as anaemia, hernias, haemorrhoids, genital and urinary tract infections, stress incontinence, and prolapse (13). We followed up the children of this cohort for their development and to determine if management at delivery or postpartum morbidities, or care-giving practices of mothers affected children's development.

## MATERIALS AND METHODS

### Study area and sample

The study conducted in Matlab, a poor rural area located about 55 km southeast of Dhaka, the capital city of Bangladesh, had several components, viz. physical, psychological and economic consequences of childbirth. Those women who became pregnant (n=4,817) over a two-year period (2007-2008) in the icddr,b service area in Matlab and their babies comprised the sample of the main study. The sample for the developmental component was smaller and was drawn from among those women examined by a physician at 6-9 weeks postpartum who had had a livebirth and whose babies were alive throughout the first year of life. Women in this cohort included those who suffered from complications (based on facility-based skilled providers' diagnosis) and a sample of those with normal vaginal births. The sample also included women who had had a caesarean section without maternal indication. The assumption guiding the sample-size was that morbidities at birth could result in maternal depression or other postpartum problems that could reduce care-giving abilities, which may, in turn, cause lower development of their children. For this descriptive study, we used a 1:2 sample-size, including 150 women with severe/less-severe obstetric complications and 300 women with a normal delivery, making a total of 450. We started enrolling mothers and their children for this component from the beginning of the study until we reached our sample-size of 488. It is to be noted that some additional children were tested before the total was reported.

### Measurements of women's status

**Socioeconomic status:** A well-established Health and Demographic Surveillance System (HDSS) has been maintained by icddr,b in Matlab since 1966. We used the latest information on education and occupation of parents and the socioeconomic status (SES) index available in the HDSS (25).

**Delivery-related information:** Women were identified, who delivered at home or were admitted for childbirth to the Matlab Hospital of icddr,b or any public or private health facility in Matlab and Chandpur. The Community Health Research Workers (CHRWs) collected information on place and mode of delivery, pregnancy outcomes, any complication during or after delivery, reason for and duration of hospitalization, and other related data. The CHRWs regularly visited the households and noted all vital events.

**Physical examination:** At 6-9 weeks postpartum, all enrolled women who had suffered from a severe complication, half of those who had suffered from a less-severe complication plus women with a caesarean section with no recorded maternal indication, and a sample of women with normal vaginal delivery (delivered either at home or facility) were invited to the nearest subcentre. A medical doctor collected information from them on morbidities relating to childbirth as perceived by them and conducted a physical examination to identify any consequences relating to their pregnancy and childbirth, e.g. vesicovaginal fistula or rectovaginal fistula, genital prolapse, urinary incontinence, dysuria, foot drop, perineal tear, pelvic inflammatory disease (PID), Sheehan's Syndrome, breast problems (abscess, mastitis), and hypertension (13).

**Anthropometry:** A paramedic measured height and weight of the women, using standard procedures at each of the 4 subcentres.

**Anaemia:** Haemoglobin level was assessed during women's visit, using a HemoCue.

**Urinary tract infection:** All women had their urine checked for pus cells and red blood cell count (RBC) as a measure of urinary tract infection (UTI), using a dipstick urine test. At the same time, approximately 10% of urine samples were sent to the Matlab Hospital Laboratory of icddr,b for routine and microscopic examinations (R/M/E) to validate dipstick results.

**Depression:** Mother's depression was measured at six weeks and six months postpartum, using Edinburgh's Postnatal Depression Scale (EPDS), which had been validated for use in Bangladesh (26). This information was collected by the Field Research Assistants (FRAs) who were trained by the principal investigator in charge of the study component (27).

The following information was collected by the FRAs who were trained by skilled research officers to collect such data:

**Care-giving practices:** A questionnaire was used for measuring care-giving practices of mothers and other family members in terms of appropriate care, nutrition, and health at 6-9 weeks postpartum based on a modified version of the Home Observation for Measurement of Environment (HOME) for infants and toddlers (28). The questionnaire had several components, viz. stimulation activities by mother and others, provision of safe environment, feeding knowledge and practices, and responsivity of the mother to her child.

### Measurements of children's status

**At 6 months:** Based on a previous study in Matlab, Bangladesh (29), 10 motor milestones were used for assessing children's motor development at six months of age. The milestones included: (a) Holds head erect and steady, (b) Lifts head and upper trunk on tummy/stomach, (c) Sits with support, (d) Picks up toy/cube, (e) Transfers object from one hand to the other, (f) Sits without support, (g) Crawling/creeping, (h) Pulls self to stand, (i) Stands assisted, and (j) Walks assisted. The sum of the milestones achieved by children was used as a proxy for their motor development at six months of age. The instrument was field-tested in another study in the same region before its use in this study. These data were collected by the Field Research Assistants who were trained by Research Officers and achieved acceptable inter-observer reliability with the trainer.

**At 12 months:** Children's mental (MDI) and psychomotor (PDI) development indices were assessed using the revised version of Bayley Scales of Infant Development (BSID-II) (30). Stimulation in the home was assessed using the family-care indicators (FCIs) (31), which were modified from the HOME (28). The comprehensive and expressive language of children was assessed through mothers' report, using a questionnaire containing 60 words that was developed by the Child Development Unit of icddr,b (32) based on MacArthur's Communicative Development Inventory (MCDI) (33).

### Statistical methods

The SPSS software (version 15) was used for data-entry and subsequent analyses. The variables were checked for normality. The language expression scores were positively skewed and were log-transformed.

**Care-giving practices:** Two care-giving scales were developed—one based on what the mother practised with the child and the other with total care-giving that the child received from the mother, other family members, and the environment. The latter also included hygiene and nutrition knowledge and practices.

**Milestones:** The milestones achieved at six months were added together to get the total number of achieved milestones at six months.

**FCI:** The FCI subscales were added together to make a ‘total FCI’ score.

The weight and height of the children were converted to z-scores, using the growth standards of the World Health Organization (WHO).

**Body mass index:** The body mass index (BMI) of mothers was calculated using the formula: weight (kg)/height (m)^2^.

**Maternal conditions:** Symptoms self-reported by those women who came for physical examination at 6-9 weeks were recorded and summed together according to the severity of the postpartum condition, and a postpartum morbidity scale was developed that represented mothers' perception of their illness. We also developed a postpartum morbidity scale based on the physical examination conducted by a physician at the same time (12).

From a child development perspective, we considered conditions, such as vesicovaginal fistula (VVF), rectovaginal fistula (RVF), third-degree genital prolapse, third-degree perineal tear, stress incontinence, peritonitis, breast abscess, mastitis, depression, moderate and severe anaemia, and BMI of <18.5 as the major and substantial morbid conditions that could affect the mother's care-giving practices and indirectly hamper children's development. Other minor postpartum morbidities, such as PID, haemorrhoids, and vaginal discharge, are chronic and persistently uncomfortable in nature and might have mild indirect effects on children's development. We, therefore, developed major and minor postpartum morbidity scales based on the above criteria to see if these affected children's development in any possible way.

We looked at the correlations of SES measures with postpartum maternal morbidities using bivariate correlations and child development measures using partial correlations controlling for the age of children which significantly correlated with developmental measures. Initially, we examined the differences in the mean values of the children's developmental outcomes by maternal conditions described above, using analysis of variance (ANOVA). Only nine children who suffered from foetal distress at birth were available for developmental testing; as the number was so small, they were dropped from analysis. To determine if major and minor morbidities (as diagnosed by a physician) affected children's development, we conducted ANOVA and multiple regression analyses controlling for the potential confounders, using the major morbidities with or without anaemia, depression, and minor morbidities.

## RESULTS

The characteristics of the sample (n=488) are presented in Table 1. Table 2 presents the prevalence of maternal morbidity conditions at 6-9 weeks postpartum. Moderate or severe anaemia was present in 35% of the women postpartum while 17% and 11% suffered from postpartum depression at six weeks and six months post-delivery respectively. Only 4% suffered from any major morbidity as detected by a physician in the postpartum period whereas 62% were diagnosed with one or more minor morbidity(ies).

**Table 1.** Characteristics of participating children and their families

Variable	Sample-size (n)	% (mean±SD)
Child characteristics		
Cephalic presentation at delivery	488	81
Gestational age (in weeks)	476	39.6±2.2
Prematurity (<37 weeks)	476	11
Sex (male)	510	49
Family characteristics		
Age (years) of mothers (6 weeks postpartum)	488	25.7±5.9
Education of mothers (<5 years of schooling)	408	19
Education of fathers (<5 years of schooling)	281	36
Occupation of mothers (housewife)	445	87
Parity (primi)	447	35
Assisted delivery (caesarean section, instrumental)	447	32

SD=Standard deviation

**Table 2.** Prevalence of self-reported and clinically-diagnosed maternal morbid conditions (n=488 unless shown in bracket)

Self-reported and clinically-diagnosed conditions	%
Self-reported symptoms	
Vesicovaginal fistula	10
Rectovaginal fistula	2.5
Uterine prolapse	15
Haemorrhoids	11
Pelvic inflammatory disease	22
Dysuria	18
Maternal depression (depressed)	
At 6 weeks postpartum (>10 points) (n=512)	17
At 6 months postpartum (>10 points) (n=503)	11
Total self-reported morbidities (n=466)	17.0±7.7*
Morbidities diagnosed by a physician	
Signs of abdominal infection	9
Stress incontinence (n=474)	1
Perineal tear	36
First-degree	15
Second-degree	20
Third-degree	1
Pelvic organ prolapse	11
Vaginal prolapse	9
First-degree uterine prolapse	2
Vaginal discharge (n=474)	30
UTI based on urine dipstick (>2+WBC) (n=485)	16
UTI based on urine test in laboratory (n=115)	7
Anaemia (Hb <11 g/dL based on HemoCue) (n=486)	35
Major morbidities	4
Minor morbidities	62

*Mean±SD; Major morbidities: presence of one or more of the following conditions (diagnosed by a doctor): Third-degree perineal tear, abdominal rebound tenderness, breast abscess, stress incontinence; Minor morbidities: presence of one or more of the following conditions (diagnosed by a doctor): Second-degree perineal tear, vaginal prolapse, haemorrhoids, vaginal discharge, UTI according to dipstick, and abdominal tenderness; SD=Standard deviation; UTI=Urinary tract infection; WBC=White blood cell

Scores of maternal care-giving practices (r=0.15, p=0.001), total care-giving practices (r=0.16, p<0.001), MDI (r=0.13, p=0.007), PDI (r=0.11, p=0.014), comprehension (r=0.12, p=0.007), expression (r=0.09, p=0.040), and the total number of milestones achieved (r=0.61, p<0.001) correlated with age of the infant. Given this, we controlled for age in further analysis of these variables. Scores of maternal care-giving practices were also associated with maternal education and other socioeconomic factors in the expected direction.

Using ANOVA controlling for age, child development measures tended to be lower in the normal-delivery group (data not shown). Moreover, we observed higher postpartum major (p=0.008) and minor (p=0.003) maternal morbidities in the normal-delivery group compared to the caesarean-section group. The normal-delivery group also came from poorer families compared to the caesarean-section group (19% vs 9% in the poorest, 21% vs 37% in the richest, p<0.001). We then conducted ANOVA controlling for age to see if the developmental outcomes differed by maternal depression, anaemia, and physician-diagnosed major and minor postpartum morbidities (Table 3). The MDI was significantly higher in the group without any major postpartum morbidity (including the group with minor morbidities) while the PDI and expression were significantly lower in the anaemic groups.

We conducted partial correlations to see if child development measures were related to the nutritional status of mothers, their SES, and postpartum maternal morbidities (Table 4). The variables that significantly correlated with the developmental measures were considered confounders and were used in multiple regression analyses. Some nutritional and socioeconomic variables were closely related to each other, e.g. education of mothers and fathers, weight-for-age, and height-for-age, etc., and we, therefore, used education of mothers and height-for-age z-scores in the multiple regression model.

We then conducted a series of multiple regressions predicting the developmental outcomes, controlling for age and other confounders and using different morbidities, i.e. major and minor morbidities, anaemia, and depression. None of the maternal postpartum morbid conditions that were related to the developmental outcomes on bivariate correlations remained significant when we controlled for the nutritional status and SES in multiple regression analyses, except for postpartum maternal anaemia. There was a trend of minor postpartum morbidities predicting language comprehension but it did not reach significance levels (B=0.7, 95% confidence interval −0.15−1.6, p=0.102). Maternal postpartum anaemia had a small but significantly negative effect on children's language expression, and the effect on comprehension approached significance. Other factors that predicted the development of children were age at test, gestational age, psychosocial stimulation at home, concurrent nutritional status, SES, and maternal education (Table 5).

## DISCUSSION

In this study, maternal anaemia measured in the postpartum period showed a small but significantly negative effect on children's language expression and an approaching effect on language comprehension. No other postpartum conditions of the mother showed an effect on children's development. However, very few mothers experienced severe postpartum morbidities, resulting in too few on which to base a conclusion with regard to such morbidities. That children of mothers with normal delivery experienced lower developmental levels is perhaps due to the fact that this group included women who delivered at home; they were also from the lower socioeconomic quintiles (34).

Maternal conditions that have been previously reported to affect children's development include low BMI in pregnancy (18,35), prenatal anaemia, and postpartum depression (21). Besides postpartum depression, we could not find any study that looked at postpartum maternal morbidities (those that related to the childbearing process) and child development.

The findings of our study showed that children's development at 12 months of age was related to psychosocial stimulation at home, their nutritional status, education of parents, SES, and care-giving practices of mothers at six weeks which is in conformity with previous findings (36).

There are no Bangladeshi standardized developmental tests for the age-group of 0-1 year but BSID-II has been used in previous studies (31,32,37-41) and appeared to be valid in this population. In the present study, the scores correlated with the SES and nutritional status of children in theoretically-expected ways. The language test was developed for children of Bangladesh based on mothers' report, which is recognized as a valid method (42) and has been used in several studies in developing countries to assess motor (43,44) and language development (45). The language inventory used in the present study had good test-retest reliability and concurrent validity (32).

**Table 3.** Developmental outcomes by physician-diagnosed maternal morbidity conditions (ANOVA controlling for age)

Morbidity group	No.	MDI	PDI	No.	Comprehension	Expression	No.	Milestones	No.	Care-giving scores by mother	Total care-giving scores
Major morbidities											
No	412	100.8±11.8**	96.1±14.2	455	18.1±7.0	4.7± 2.4	403	6.2± 1.7	417	8.5±2.7	47.9±8.3
Yes	16	94.3±10.7	92.4±14.7	19	18.2±5.3	3.5±1.2	18	6.8± 1.7	17	8.2±2.2	46.3±8.9
Minor morbidities											
No	165	100.9±12.3	96.1±15.4	181	18.2±6.5	4.8±2.3	155	6.2±1.7	162	8.4±2.8	48.4±8.4
Yes	261	100.3±11.5	96.2±13.3	290	18.1±7.1	4.6±2.5	264	6.2±1.8	271	8.4±2.6	47.4±8.3
Depression											
No	368	100.6±12.1	96.0±14.4	423	18.1±6.9	4.7±2.5	368	6.2±1.8*	390	8.5±2.7*	48.3±8.4
Yes	82	99.7±10.8	95.4±12.6	89	17.5±7.1	4.4±2.4	85	6.0±1.6	80	7.9±2.6	45.4±8.7
Anaemia											
No	283	101.0±12.1	97.2±13.8***	314	18.3±6.7	4.8±2.4***	273	6.2±1.7	287	8.5±2.8	47.8±8.6
Yes	158	99.8±11.2	93.7±14.5	172	17.6±7.3	4.3±2.4	158	6.2±1.9	158	8.2±2.6	47.9±8.0

Major morbidities: presence of one or more of the following conditions (diagnosed by a doctor): Third-degree perineal tear, abdominal rebound tenderness, breast abscess, and stress incontinence. Minor morbidities: presence of one or more of the following conditions (diagnosed by a doctor): Second-degree perineal tear, vaginal prolapse, haemorrhoids, vaginal discharge, UTI according to dipstick, and abdominal tenderness

*p<0.1

**p<0.05

***p<0.01; ANOVA=Analysis of variance; MDI=Mental development index; PDI=Psychomotor development index; UTI=Urinary tract infection

In a 2007 series of articles published by the *Lancet*, it was reported that over 200 million children did not achieve their developmental potentials (1,2,46). In these articles, iron deficiency was identified as one of the major three causes of developmental delay. It is now established that iron-deficiency anaemia in infants aged 6-24 months is a risk factor for poorer cognitive, motor, social-emotional and neurophysiologic development, and the developmental effects of prenatal iron deficiency are now under consideration (47). Maternal anaemia early in pregnancy has been reported to result in low birthweight subsequent to preterm delivery and low Apgar scores at birth (17), infant respiratory health outcomes in terms of wheezing in the first year of life (48), and irritability in the infant on the Brazelton score (49). In an animal study, rhesus monkey infants which were prenatally deprived of iron showed a lower spontaneous activity level and inhibitory response to novel environments while postnatally-deprived infants had poorer object concept task performance and greater emotionality compared to controls (50). Maternal iron deficiency measured during pregnancy has been reported to affect maternal responsivity (51) while postpartum maternal iron-deficiency anaemia was associated with poorer mother-child interaction (20) and lower maternal responsivity and delayed infant development at nine months of age even after correction of anaemia (52). Antenatal iron and folic acid supplementation was associated with improved mental and motor function of children at 7-9 years of age in rural Nepal (53). In addition, the severity of anaemia in young women affected their processing speed and cognitive function (54) and may, therefore, also affect their childrearing abilities.

### Limitation

A major limitation of the study was that we did not have a baseline of morbidities before delivery, i.e. pregnancy-related data; hence, maternal anaemia that was detected postpartum may have been there during pregnancy as well.

### Conclusions

Attention should be paid to the correction of maternal prenatal and postnatal anaemia, especially in the poor and underprivileged population as this affects the next generation's language development. Further research with sufficient sample-size for individual morbidities is required to determine if delivery-related and postpartum maternal morbidities, including postpartum anaemia, affect children's development.

**Table 4.** Correlation of developmental variables controlling for age at the time of assessment with socioeconomic factors, nutritional status, and postpartum maternal morbidity

Parameter	MDI	PDI	Compre-hension	Expression	Milestone	Care-giving scores by mother	Total care-giving scores
Characteristics of children							
Gestational age (weeks)	0.08*	0.12***	0.08*	0.01	0.14***	0.02	0.02
HAZ	0.10**	0.24***	0.21***	0.15***	0.19***	0.06	0.08
WAZ	0.05	0.16***	0.17***	0.19***	0.18***	0.13***	0.10**
WHZ	0.02	0.05**	0.09*	0.16***	0.12**	0.14***	0.08
MUAC	0.04	0.06	0.19***	0.17***	0.10*	0.09*	0.06
Head-circumference	0.10**	0.11**	0.12**	0.08*	0.05	0.14***	0.09*
Characteristics of families							
Total FCI	0.11**	0.13***	0.52***	0.38***	0.02	0.30***	0.27***
BMI of mothers	0.07	0.14***	0.16***	0.08*	0.14***	0.13***	0.05
Age of mothers	0.08	0.005	0.03	0.003	0.05	-0.02	-0.09*
Education of mothers	0.09*	0.20***	0.23***	0.26***	0.14***	0.13**	0.11**
Occupation of mothers	0.08	0.07	0.08*	0.09**	0.03	0.12**	0.11**
MUAC of mothers	0.04	0.15**	0.18***	0.12**	0.12**	0.12**	0.04
Parity	-0.03	-0.02	0.07	-0.06	0.04	-0.02	-0.10**
Education of fathers	0.09	0.19***	0.24***	0.23***	0.17***	0.12*	0.16**
Socioeconomic status	0.06	0.19***	0.23***	0.23***	0.12**	0.12**	0.15***
Maternal morbid conditions							
Maternal anaemia	-0.04	-0.13***	-0.06	-0.12**	-0.04	-0.07	0.01
Maternal depression–6 weeks	-0.04	-0.05	-0.06	-0.03	-0.08*	-0.07	-0.16***
Self-reported morbidity scale	-0.09*	-0.09*	-0.10**	-0.13***	-0.06	-0.11**	-0.10**
Total morbidity score on examination	-0.02	-0.13**	-0.02	-0.15***	-0.01	-0.03	-0.05
Major morbidities	-0.10**	-0.05	0.01	-0.10**	0.04	-0.02	-0.04
Minor morbidities	-0.02	-0.04	-0.02	-0.09*	0.02	0.000	-0.06

*p<0.1

**p<0.05

***p<0.01

BMI=Body mass index; FCI=Family-care indicator; HAZ=Height-for-age; MDI=Mental development index; MUAC=Mid-upper arm-circumference; PDI=Psychomotor development index; WAZ=Weight-for-age; WHZ=Weight-for-height

**Table 5.** Regression coefficients and 95% confidence interval for the effect of mothers' postpartum anaemia on developmental outcomes

Parameter	MDI (n=431)	p ratio	PDI (n=321)	p value	Comprehension (n=336)	p value	Expression (n=336)	p value	Milestone (n=288)	p value
Child's age	-1.2 (-2.4,-0.02)	0.046	2.4 (0.7,4.1)	0.005	1.7 (0.8, 2.5)	<0.001	0.04 (0.02,0.06)	0.001	1.4 (1.2,1.6)	<0.001
FCI	0.3 (0.01,0.7)	0.044			1.0 (0.8,1.2)	<0.001	0.02 (0.01,0.02)	<0.001	NS	
HAZ	NS		1.6 (0.2,3.0)	0.023	1.0 (0.4,1.6)	0.001	NS		0.15 (0.008,0.3)	0.039
Education of mother			NS		NS		0.008 (0.002, 0.01)	0.009	NS	
SES	-		1.6 (0.6,2.6)	0.002	NS		NS		0.12 (0.01,0.2)	0.027
Gestational age	-		0.7 (-0.01,1.3)	0.054	-		-		0.08 (0.01,0.1)	0.024
Maternal anaemia	-1.2 (-3.5,1.0)	0.280	-2.1 (-5.1,0.9)	0.170	-1.2 (-2.5,0.2)	0.084	-0.04 (-0.07,-0.002)	0.038	-0.04 (-0.4,0.3)	0.791
R^2^	0.02		0.08		0.28		0.18		0.47	

Models: Mental development index: Step 1: Child's age at test (entered); Step 2: Head-circumference, HAZ, and FCI at 12 months (offered); Step 3: Maternal anaemia (0=Hb ≥11 g/dL, 1=Hb <11 g/dL) (entered) Psychomotor development index: Step 1: Child's age at test (entered); Step 2: Gestational age at birth, head-circumference, HAZ, and FCI at 12 months, mothers' MUAC, education, and SES quintile (offered); Step 3: Maternal anaemia (0=Hb ≥11 g/dL, 1=Hb <11 g/dL) (entered) Language comprehension: Step 1: Child's age at test (entered), Step 2: MUAC, HAZ, and FCI at 12 months and mothers' MUAC, education, and SES quintile (offered), Step 3: Maternal anaemia (0=Hb ≥11 g/dL, 1=Hb <11 g/dL) (entered) Language expression: Step 1: Child's age at test (entered), Step 2: MUAC, HAZ, and FCI at 12 months and mothers' MUAC, education, and SES quintile (offered), Step 3: Maternal anaemia (0=Hb ≥11 g/dL, 1=Hb <11 g/dL) (entered) Milestones achieved: Step 1: Child's age at test (entered), Step 2: Gestational age at birth and HAZ at 12 months, mothers' BMI and education, and SES quintile (offered), Step 3: Maternal anaemia (0=Hb ≥11g/dL, 1=Hb <11 g/dL) (entered) BMI=Body mass index; FCI=Family-care indicators; HAZ=Height-for-age; MDI=Mental development index; MUAC=Mid-upper arm-circumference; NS=Not significant; PDI=Psychomotor development index; SES=Socioeconomic status

## ACKNOWLEDGEMENTS

This study was funded by USAID (United States Agency for International Development) (Grant number GHS-A00-0300019-00). icddr,b acknowledges with gratitude the commitment of USAID to its research efforts.

We are grateful to the mothers and children who participated in this study. We also acknowledge the services of members of the Child Development Unit as well as those of Matlab Health and Demographic Surveillance System (HDSS) of icddr,b for their continued support and cooperation.

## References

[1] Grantham-McGregor S, Cheung YB, Cueto S, Glewwe P, Richter L, Strupp B, International Child Development Steering Group (2007). Developmental potential in the first 5 years for children in developing countries. Lancet.

[2] Walker SP, Wachs TD, Gardner JM, Lozoff B, Wasserman GA, Pollitt E (2007). Child development: risk factors for adverse outcomes in developing countries. Lancet.

[3] Black RE, Allen LH, Bhutta ZA, Caulfield LE, de Onis M, Ezzati M (2008). Maternal and child undernutrition: global and regional exposures and health consequences. Lancet.

[4] Huddy C, Johnson A, Hope PL (2001). Educational and behavioural problems in babies of 32-35 weeks gestation. Arch Dis Child Fetal Neonatal Ed.

[5] Wang ML, Dorer DJ, Fleming MP, Catlin EA (2004). Clinical outcomes of near-term infants. Pediatrics.

[6] Petrou S (2005). The economic consequences of pretermbirth during the first 10 years of life. BJOG.

[7] Petrou S, Mehta Z, Hockley C, Cook-Mozaffari P, Henderson J, Goldacre M (2003). The impact of preterm birth on hospital inpatient admissions and costs during the first 5 years of life. Pediatrics.

[8] Victora CG, Adair L, Fall C, Hallal PC, Martorell R, Richter L (2008). Maternal and child undernutrition: consequences for adult health and human capital. Lancet.

[9] Grantham-McGregor S, Ani C (2001). A review of studies on the effect of iron deficiency on cognitive development in children. J Nutr.

[10] Gülmezoglu AM, Say L, Betrán AP, Villar J, Piaggio G (2004). WHO systematic review of maternal mortality and morbidity: methodological issues and challenges. BMC Med Res Methodol.

[11] Razzaque A, Da Vanzo J, Rahman M, Gausia K, Hale L, Khan MA (2005). Pregnancy spacing and maternal morbidity in Matlab, Bangladesh. Int J Gynecol Obstet.

[12] Huda FA, Ahmed A, Dasgupta SK, Jahan M, Ferdous J, Koblinsky M (2012). Profile of maternal and foetal complications during labour and delivery among women giving birth in hospitals in Matlab and Chandpur, Bangladesh. J Health Popul Nutr.

[13] Ferdous J, Ahmed A, Dasgupta SK, Jahan M, Huda FA, Ronsmans C (2012). Occurrence and determinants of postpartum maternal morbidities and disabilities among women in Matlab, Bangladesh. J Health Popul Nutr.

[14] Kusiako T, Ronsmans C, Van der Paal L (2000). Perinatal mortality attributable to complications of childbirth in Matlab, Bangladesh. Bull World Health Organ.

[15] Deregnier RA, Nelson CA, Thomas KM, Wewerka S, Georgieff MK (2000). Neurophysiologic evaluation of auditory recognition memory in healthy newborn infants and infants of diabetic mothers. J Pediatr.

[16] van der Pol MC, Hadders-Algra M, Huisjes HJ, Touwen BC (1991). Antiepileptic medication in pregnancy: late effects on the children's central nervous system development. Am J Obstet Gynecol.

[17] Allen LH (2000). Anemia and iron deficiency: effects on pregnancy outcome. Am J Clin Nutr.

[18] Kilbride J, Baker TG, Parapia LA, Khoury SA, Shuqaidef SW, Jerwood D (1999). Anaemia during pregnancy as a risk factor for iron-deficiency anaemia in infancy: a case-control study in Jordan. Int J Epidemiol.

[19] Lozoff B, Grantham-McGregor SM (1998). Explanatory mechanisms for poorer development in iron-deficient anemic infants. Nutrition, health, and child development: research advances and policy recommendations.

[20] Murray-Kolb LE, Beard JL (2009). Iron deficiency and child and maternal health. Am J Clin Nutr.

[21] Black MM, Baqui AH, Zaman K, McNary SW, Le K, Arifeen SE (2007). Depressive symptoms among rural Bangladeshi mothers: implications for infant development. J Child Psychol Psychiatry.

[22] Khadem N, Khadivzadeh T (2010). The intelligence quotient of school aged children delivered by cesarean section and vaginal delivery. Iran J Nurs Midwifery Res.

[23] Eide MG, Øyen N, Skjaerven R, Irgens LM, Bjerkedal T, Nilsen ST (2005). Breech delivery and intelligence: a population-based study of 8,738 breech infants. Obstet Gynecol.

[24] Seidman DS, Laor A, Gale R, Stevenson DK, Mashiach S, Danon YL (1991). Long-term effects of vacuum and forceps deliveries. Lancet.

[25] International Centre for Diarrhoeal Disease Research, Bangladesh (2009). Health and demographic surveillance system, Matlab. V. 41. Registration of health and demographic events, 2007.

[26] Gausia K, Hamadani JD, Islam MM, Ali M, Algin S, Yunus M (2007). Bangla translation, adaptation and piloting of Edinburgh Postnatal Depression Scale. Bangladesh Med Res Counc Bull.

[27] Gausia K, Ryder D, Ali M, Fisher C, Moran A, Koblinsky M (2012). Obstetric complications and psychological well-being: experiences of Bangladeshi women during pregnancy and childbirth. J Health Popul Nutr.

[28] Bradley RH (1993). Children's home environments, health, behavior, and intervention efforts: a review using the HOME inventory as a marker measure. Genet Soc Gen Psychol Monogr.

[29] Tofail F Effect of food and micronutrient supplementation during pregnancy on subsequent development of infants in Bangladesh: a randomized trial. London, Institute of Child Health, University College London, 2006. 271 p. (Monograph of PhD thesis)..

[30] Bayley N (1993). Bayley scales of infant development.

[31] Hamadani JD, Tofail F, Hilaly A, Huda SN, Engle P, Grantham-McGregor SM (2010). Use of family care indicators and their relationship with child development in Bangladesh. J Health Popul Nutr.

[32] Hamadani JD, Baker-Henningham H, Tofail F, Mehrin F, Huda SN, Grantham-McGregor SM (2010). Validity and reliability of mothers' reports of language development in 1-year-old children in a large-scale survey in Bangladesh. Food Nutr Bull.

[33] Fenson L, Dale PS, Reznick JS, Bates E, Thal DJ, Pethick SJ (1994). Variability in early communicative development. Monogr Soc Res Child Dev.

[34] Hoque ME, Powell-Jackson T, Dasgupta SK, Chowdhury ME, Koblinsky M (2012). Costs of maternal health-related complications in Bangladesh. J Health Popul Nutr.

[35] Tofail F, Persson LA, El Arifeen S, Hamadani JD, Mehrin F, Ridout D (2008). Effects of prenatal food and micronutrient supplementation on infant development: a randomized trial from the Maternal and Infant Nutrition Interventions, Matlab (MINIMat) study. Am J Clin Nutr.

[36] Walker SP, Wachs TD, Grantham-McGregor S, Black MM, Nelson CA, Huffman SL (2011). Inequality in early childhood: risk and protective factors for early child development. Lancet.

[37] Hamadani JD, Huda SN, Khatun F, Grantham-McGregor SM (2006). Psychosocial stimulation improves the development of undernourished children in rural Bangladesh. J Nutr.

[38] Hamadani JD, Fuchs GJ, Osendarp SJ, Khatun F, Huda SN, Grantham-McGregor SM (2001). Randomized controlled trial of the effect of zinc supplementation on the mental development of Bangladeshi infants. Am J Clin Nutr.

[39] Hamadani JD, Fuchs GJ, Osendarp SJ, Huda SN, Grantham-McGregor SM (2002). Zinc supplementation during pregnancy and effects on mental development and behaviour of infants: a follow-up study. Lancet.

[40] Tofail F, Kabir I, Hamadani JD, Chowdhury F, Yesmin S, Mehreen F (2006). Supplementation of fish-oil and soy-oil during pregnancy and psychomotor development of infants. J Health Popul Nutr.

[41] Black MM, Baqui AH, Zaman K, Ake Persson L, El Arifeen S, Le K (2004). Iron and zinc supplementation promote motor development and exploratory behavior among Bangladeshi infants. Am J Clin Nutr.

[42] Fenson L, Bates E, Dale P, Goodman J, Reznick JS, Thal D (2000). Measuring variability in early child language: don't shoot the messenger. Child Dev.

[43] Cheung YB, Yip PS, Karlberg JP (2001). Fetal growth, early postnatal growth and motor development in Pakistani infants. Int J Epidemiol.

[44] Pollitt E, Durnin JV, Husaini M, Jahari A (2000). Effects of an energy and micronutrient supplement on growth and development in undernourished children in Indonesia: methods. Eur J Clin Nutr.

[45] Stoltzfus RJ, Kvalsvig JD, Chwaya HM, Montresor A, Albonico M, Tielsch JM (2001). Effects of iron supplementation and anthelmintic treatment on motor and language development of preschool children in Zanzibar: double blind, placebo controlled study. BMJ.

[46] Engle PL, Black MM, Behrman JR, Cabral de Mello M, Gertler PJ, Kapiriri L (2007). (on behalf of International Child Development Steering Group). Strategies to avoid the loss of developmental potential in more than 200 million children in the developing world. Lancet.

[47] Lozoff B (2007). Iron deficiency and child development. Food Nutr Bull.

[48] Triche EW, Lundsberg LS, Wickner PG, Belanger K, Leaderer BP, Bracken MB (2011). Association of maternal anemia with increased wheeze and asthma in children. Ann Allergy Asthma Immunol.

[49] Vaughn J, Brown J, Carter JP (1986). The effects of maternal anemia on infant behavior. J Natl Med Assoc.

[50] Golub MS, Hogrefe CE, Germann SL, Capitanio JP, Lozoff B (2006). Behavioral consequences of developmental iron deficiency in infant rhesus monkeys. Neurotoxicol Teratol.

[51] Wachs TD, Kanashiro HC, Gurkas P (2008). Intra-individual variability in infancy: structure, stability, and nutritional correlates. Dev Psychobiol.

[52] Perez EM, Hendricks MK, Beard JL, Murray-Kolb LE, Berg A, Tomlinson M (2005). Mother-infant interactions and infant development are altered by maternal iron deficiency anemia. J Nutr.

[53] Christian P, Murray-Kolb LE, Khatry SK, Katz J, Schaefer BA, Cole PM (2010). Prenatal micronutrient supplementation and intellectual and motor function in early school-aged children in Nepal. JAMA.

[54] Murray-Kolb LE, Beard JL (2007). Iron treatment normalizes cognitive functioning in young women. Am J Clin Nutr.

